# (±)-5-Ethyl-2-(4-isopropyl-4-methyl-5-oxo-4,5-dihydro-1*H*-imidazol-2-yl)nicotinic acid

**DOI:** 10.1107/S1600536808007411

**Published:** 2008-05-03

**Authors:** Wei Dai, Da-Wei Fu

**Affiliations:** a Ordered Matter Science Research Center, College of Chemistry and Chemical Engineering, Southeast University, Nanjing 210096, People’s Republic of China

## Abstract

In the title compound, C_15_H_19_N_3_O_3_, owing to an intra­molecular O—H⋯N hydrogen bond, the pyridine and imidazole rings are nearly coplanar and are twisted from each other by a dihedral angle of only 0.92 (9)°. The mol­ecules are linked through inter­molecular N—H⋯O hydrogen bonding, forming an infinite chain parallel to the *b* axis.

## Related literature

For usages of nicotinic acid and imidazole in coordination chemistry and medicinal chemistry, see: Liu *et al.* (2005 ▶); Zhao *et al.* (2007 ▶); He *et al.* (2005 ▶); Boovanahalli *et al.* (2007 ▶); Song *et al.* (2006 ▶).
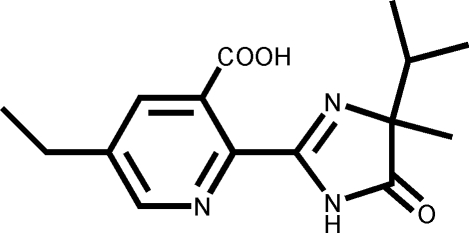


## Experimental

### 

#### Crystal data

C_15_H_19_N_3_O_3_
*M* *_r_* = 289.33
Monoclinic, 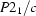

*a* = 12.6916 (15) Å
*b* = 16.0748 (17) Å
*c* = 7.3801 (8) Å
β = 100.213 (7)°
*V* = 1481.8 (3) Å^3^
*Z* = 4
Mo *K*α radiation
μ = 0.09 mm^−1^
*T* = 293 (2) K
0.25 × 0.25 × 0.20 mm

#### Data collection

Rigaku Mercury2 diffractometer
Absorption correction: multi-scan (*CrystalClear*; Rigaku, 2005 ▶) *T*_min_ = 0.978, *T*_max_ = 0.988
15016 measured reflections
3357 independent reflections
2413 reflections with *I* > 2σ(*I*)
*R*_int_ = 0.045

#### Refinement

*R*[*F* ^2^ > 2σ(*F* ^2^)] = 0.051
*wR*(*F* ^2^) = 0.136
*S* = 1.03
3357 reflections
195 parameters
H-atom parameters constrained
Δρ_max_ = 0.20 e Å^−3^
Δρ_min_ = −0.20 e Å^−3^

### 

Data collection: *CrystalClear* (Rigaku, 2005 ▶); cell refinement: *CrystalClear*; data reduction: *CrystalClear*; program(s) used to solve structure: *SHELXS97* (Sheldrick, 2008 ▶); program(s) used to refine structure: *SHELXL97* (Sheldrick, 2008 ▶); molecular graphics: *ORTEPIII* (Burnett & Johnson, 1996 ▶), *ORTEP-3 for Windows* (Farrugia, 1997 ▶) and *PLATON* (Spek, 2003 ▶); software used to prepare material for publication: *SHELXL97*.

## Supplementary Material

Crystal structure: contains datablocks I, global. DOI: 10.1107/S1600536808007411/dn2325sup1.cif

Structure factors: contains datablocks I. DOI: 10.1107/S1600536808007411/dn2325Isup2.hkl

Additional supplementary materials:  crystallographic information; 3D view; checkCIF report

## Figures and Tables

**Table 1:** Hydrogen-bond geometry (Å, °)

*D*—H⋯*A*	*D*—H	H⋯*A*	*D*⋯*A*	*D*—H⋯*A*
O1—H1⋯N2	0.82	1.68	2.4984 (18)	178
N3—H3⋯O2^i^	0.86	2.10	2.9330 (19)	162

Symmetry code: (i) 
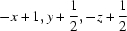
.

**Fig. 1.**
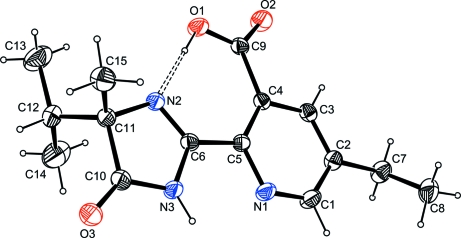
Molecular view of the title compound with the atom-labelling scheme. Displacement ellipsoids are drawn at the 30% probability level. H atoms are represented as small spheres of arbitrary radii.H bond is shown as dashed line

**Fig. 2.**
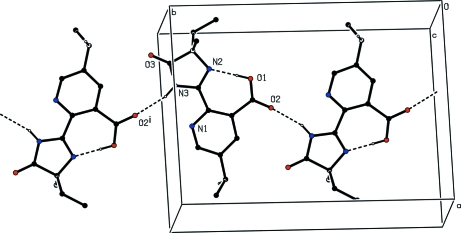
Partial packing view of the title compound sgowing the formation of the chain parallel to the b axis. H bonds are shown as dashed lines. H atoms not involved in hydrogen bondins have been omitted for clarty.[Symmetry code: (i) 1-x, 1/2+y, 1/2-z]

## supplementary crystallographic information

### Comment

The nicotinic acid and the imidazole group have found a wide range of applications in coordination chemistry as ligands, in medicinal chemistry and materials science (Liu *et al.* 2005; Zhao *et al.* 2007;He *et al.* 2005; Boovanahalli *et al.* 2007; Song *et al.* 2006). We report here the crystal structure of the title compound, C_15_H_19_N_3_O_3_.

Owing to an intramolecular O1-H1···N2 hydrogen, the pyridine and the imidazole rings are nearly planar, they are only twisted to each other by a dihedral angle of 0.91 (9) . In the imidazole ring, the C6=N2 bond distance of 1.282 (4) Å conforms to the value for a double bond, while the C11—N2 bond length of 1.472 (4) Å conforms to the value for a single bond. To the carboxyl group, the C9=O2 bond distance of 1.212 (4) Å conforms to the value for a double bond, while the C9—O1 bond length of 1.298 (4) Å conforms to the value for a single bond.

The molecules are linked through intermolecular N3-H3···O2 hydrogen bond forming an infinite chain parallel to the b axis. (Table 1 and Fig. 2).

### Experimental

5-ethyl-2-(4-isopropyl-4-methyl-5-oxo-4,5-dihydro-1*H*-imidazol-2-yl)nicotinic acid (3 mmol) was dissolved in ethanol (20 ml) and evaporated in the air affording colorless block crystals of this compound suitable for X-ray analysis were obtained.

### Refinement

All H atoms attached to C, N and O atoms were fixed geometrically and treated as riding with C–H = 0.98 Å (methine), 0.97Å(methylene), 0.96Å (methyl) and N–H= 0.86Å or O–H= 0.82 Å with U_iso_(H) = 1.2U_eq_(C, N) or U_iso_(H) = 1.5U_eq_(O, methyl).

### Crystal data

**Table:**

C_15_H_19_N_3_O_3_	*F*_000_ = 616
*M**_r_* = 289.33	*D*_x_ = 1.297 Mg m^−^^3^
Monoclinic, *P*2_1_/*c*	Mo *K*α radiation λ = 0.71073 Å
Hall symbol: -P 2ybc	Cell parameters from 2685 reflections
*a* = 12.6916 (15) Å	θ = 3.0–27.5º
*b* = 16.0748 (17) Å	µ = 0.09 mm^−^^1^
*c* = 7.3801 (8) Å	*T* = 293 (2) K
β = 100.213 (7)º	Block, colorless
*V* = 1481.8 (3) Å^3^	0.25 × 0.25 × 0.20 mm
*Z* = 4	

### Data collection

**Table:**

Rigaku Mercury2 (2x2 bin mode) diffractometer	3357 independent reflections
Radiation source: fine-focus sealed tube	2413 reflections with *I* > 2σ(*I*)
Monochromator: graphite	*R*_int_ = 0.045
Detector resolution: 13.6612 pixels mm^-1^	θ_max_ = 27.4º
*T* = 293(2) K	θ_min_ = 3.0º
ω scans	*h* = −16→16
Absorption correction: Multi-scan(CrystalClear; Rigaku, 2005)	*k* = −20→20
*T*_min_ = 0.978, *T*_max_ = 0.988	*l* = −9→9
15016 measured reflections	

### Refinement

**Table:**

Refinement on *F*^2^	Secondary atom site location: difference Fourier map
Least-squares matrix: full	Hydrogen site location: inferred from neighbouring sites
*R*[*F*^2^ > 2σ(*F*^2^)] = 0.051	H-atom parameters constrained
*wR*(*F*^2^) = 0.136	*w* = 1/[σ^2^(*F*_o_^2^) + (0.0611*P*)^2^ + 0.3777*P*] where *P* = (*F*_o_^2^ + 2*F*_c_^2^)/3
*S* = 1.03	(Δ/σ)_max_ < 0.001
3357 reflections	Δρ_max_ = 0.20 e Å^−^^3^
195 parameters	Δρ_min_ = −0.20 e Å^−^^3^
Primary atom site location: structure-invariant direct methods	Extinction correction: none

### Special details

**Table:**

Geometry. All esds (except the esd in the dihedral angle between two l.s. planes) are estimated using the full covariance matrix. The cell esds are taken into account individually in the estimation of esds in distances, angles and torsion angles; correlations between esds in cell parameters are only used when they are defined by crystal symmetry. An approximate (isotropic) treatment of cell esds is used for estimating esds involving l.s. planes.
Refinement. Refinement of *F*^2^ against ALL reflections. The weighted *R*-factor *wR* and goodness of fit *S* are based on *F*^2^, conventional *R*-factors *R* are based on *F*, with *F* set to zero for negative *F*^2^. The threshold expression of *F*^2^ > σ(*F*^2^) is used only for calculating *R*-factors(gt) *etc*. and is not relevant to the choice of reflections for refinement. *R*-factors based on *F*^2^ are statistically about twice as large as those based on *F*, and *R*- factors based on ALL data will be even larger.

### Fractional atomic coordinates and isotropic or equivalent isotropic displacement parameters (Å^2^)

**Table:**

	*x*	*y*	*z*	*U*_iso_*/*U*_eq_	
N1	0.56210 (12)	0.92867 (8)	0.3216 (2)	0.0442 (4)	
N2	0.31126 (10)	0.86524 (8)	0.06785 (19)	0.0358 (3)	
N3	0.38168 (11)	0.99041 (8)	0.1425 (2)	0.0419 (4)	
H3	0.4293	1.0259	0.1901	0.050*	
O1	0.36104 (10)	0.71477 (7)	0.09236 (19)	0.0484 (4)	
H1	0.3450	0.7642	0.0817	0.073*	
O2	0.48765 (11)	0.63866 (7)	0.2486 (2)	0.0590 (4)	
O3	0.24362 (11)	1.07812 (8)	0.0169 (2)	0.0626 (4)	
C1	0.65661 (14)	0.90689 (11)	0.4182 (3)	0.0465 (5)	
H1A	0.7023	0.9491	0.4702	0.056*	
C2	0.69167 (13)	0.82543 (11)	0.4465 (2)	0.0385 (4)	
C3	0.62079 (13)	0.76450 (10)	0.3712 (2)	0.0354 (4)	
H3A	0.6409	0.7090	0.3883	0.042*	
C4	0.51990 (12)	0.78314 (9)	0.2704 (2)	0.0304 (3)	
C5	0.49432 (12)	0.86849 (9)	0.2486 (2)	0.0328 (4)	
C6	0.39348 (13)	0.90513 (9)	0.1502 (2)	0.0337 (4)	
C7	0.80073 (15)	0.80620 (13)	0.5550 (3)	0.0508 (5)	
H7A	0.8117	0.7465	0.5559	0.061*	
H7B	0.8035	0.8240	0.6813	0.061*	
C8	0.89069 (16)	0.84795 (14)	0.4789 (3)	0.0624 (6)	
H8A	0.8871	0.8321	0.3525	0.094*	
H8B	0.9583	0.8309	0.5492	0.094*	
H8C	0.8837	0.9072	0.4868	0.094*	
C9	0.45278 (13)	0.70696 (10)	0.2013 (2)	0.0366 (4)	
C10	0.28196 (14)	1.00956 (10)	0.0468 (3)	0.0429 (4)	
C11	0.22886 (13)	0.92565 (10)	−0.0124 (2)	0.0373 (4)	
C12	0.12537 (15)	0.91484 (12)	0.0676 (3)	0.0492 (5)	
H12	0.0754	0.9583	0.0133	0.059*	
C13	0.0711 (2)	0.83164 (17)	0.0180 (4)	0.0788 (8)	
H13A	0.1161	0.7876	0.0757	0.118*	
H13B	0.0592	0.8244	−0.1131	0.118*	
H13C	0.0038	0.8303	0.0602	0.118*	
C14	0.1441 (2)	0.92685 (19)	0.2749 (3)	0.0857 (9)	
H14A	0.0772	0.9224	0.3177	0.128*	
H14B	0.1745	0.9809	0.3050	0.128*	
H14C	0.1924	0.8849	0.3329	0.128*	
C15	0.21042 (17)	0.91982 (12)	−0.2218 (3)	0.0527 (5)	
H15A	0.2770	0.9283	−0.2638	0.079*	
H15B	0.1600	0.9617	−0.2737	0.079*	
H15C	0.1827	0.8658	−0.2596	0.079*	

### Atomic displacement parameters (Å^2^)

**Table:**

	*U*^11^	*U*^22^	*U*^33^	*U*^12^	*U*^13^	*U*^23^
N1	0.0384 (8)	0.0285 (7)	0.0620 (10)	−0.0038 (6)	−0.0019 (7)	−0.0006 (7)
N2	0.0334 (8)	0.0269 (7)	0.0458 (8)	0.0002 (5)	0.0031 (6)	−0.0001 (6)
N3	0.0331 (8)	0.0247 (7)	0.0644 (10)	−0.0015 (5)	−0.0008 (7)	−0.0006 (6)
O1	0.0437 (7)	0.0250 (6)	0.0718 (9)	−0.0017 (5)	−0.0033 (6)	−0.0029 (6)
O2	0.0513 (8)	0.0237 (6)	0.0970 (11)	0.0032 (5)	−0.0003 (7)	0.0047 (6)
O3	0.0492 (8)	0.0285 (7)	0.1029 (12)	0.0087 (6)	−0.0057 (8)	0.0011 (7)
C1	0.0397 (10)	0.0354 (9)	0.0593 (12)	−0.0075 (7)	−0.0049 (8)	0.0004 (8)
C2	0.0355 (9)	0.0396 (9)	0.0395 (9)	−0.0003 (7)	0.0046 (7)	0.0054 (7)
C3	0.0386 (9)	0.0292 (8)	0.0400 (9)	0.0040 (7)	0.0113 (7)	0.0051 (7)
C4	0.0324 (8)	0.0261 (7)	0.0344 (8)	0.0009 (6)	0.0105 (6)	0.0024 (6)
C5	0.0317 (8)	0.0266 (8)	0.0403 (9)	−0.0008 (6)	0.0072 (7)	0.0011 (7)
C6	0.0349 (9)	0.0251 (8)	0.0421 (9)	−0.0004 (6)	0.0091 (7)	0.0006 (7)
C7	0.0420 (10)	0.0496 (11)	0.0555 (12)	0.0018 (8)	−0.0059 (9)	0.0090 (9)
C8	0.0375 (11)	0.0660 (14)	0.0808 (16)	0.0043 (9)	0.0022 (10)	−0.0005 (11)
C9	0.0375 (9)	0.0255 (8)	0.0482 (10)	0.0001 (7)	0.0112 (8)	−0.0016 (7)
C10	0.0386 (10)	0.0300 (9)	0.0593 (11)	0.0029 (7)	0.0064 (8)	0.0006 (8)
C11	0.0334 (9)	0.0291 (8)	0.0476 (10)	0.0020 (6)	0.0021 (7)	0.0004 (7)
C12	0.0350 (10)	0.0503 (11)	0.0615 (12)	−0.0035 (8)	0.0063 (8)	−0.0040 (9)
C13	0.0650 (15)	0.0823 (18)	0.0907 (19)	−0.0356 (13)	0.0180 (13)	−0.0154 (14)
C14	0.0713 (17)	0.122 (2)	0.0717 (17)	−0.0306 (15)	0.0344 (13)	−0.0326 (16)
C15	0.0595 (13)	0.0493 (11)	0.0468 (11)	0.0068 (9)	0.0029 (9)	0.0032 (9)

### Geometric parameters (Å, °)

**Table:**

N1—C1	1.329 (2)	C7—H7A	0.9700
N1—C5	1.342 (2)	C7—H7B	0.9700
N2—C6	1.282 (2)	C8—H8A	0.9600
N2—C11	1.472 (2)	C8—H8B	0.9600
N3—C10	1.370 (2)	C8—H8C	0.9600
N3—C6	1.379 (2)	C10—C11	1.536 (2)
N3—H3	0.8600	C11—C15	1.524 (3)
O1—C9	1.298 (2)	C11—C12	1.543 (3)
O1—H1	0.8200	C12—C14	1.518 (3)
O2—C9	1.2118 (19)	C12—C13	1.519 (3)
O3—C10	1.209 (2)	C12—H12	0.9800
C1—C2	1.387 (2)	C13—H13A	0.9600
C1—H1A	0.9300	C13—H13B	0.9600
C2—C3	1.378 (2)	C13—H13C	0.9600
C2—C7	1.503 (2)	C14—H14A	0.9600
C3—C4	1.395 (2)	C14—H14B	0.9600
C3—H3A	0.9300	C14—H14C	0.9600
C4—C5	1.412 (2)	C15—H15A	0.9600
C4—C9	1.527 (2)	C15—H15B	0.9600
C5—C6	1.477 (2)	C15—H15C	0.9600
C7—C8	1.515 (3)		
			
C1—N1—C5	118.60 (14)	O2—C9—O1	120.50 (15)
C6—N2—C11	108.73 (13)	O2—C9—C4	118.47 (15)
C10—N3—C6	109.15 (14)	O1—C9—C4	121.02 (13)
C10—N3—H3	125.4	O3—C10—N3	127.14 (17)
C6—N3—H3	125.4	O3—C10—C11	127.32 (16)
C9—O1—H1	109.5	N3—C10—C11	105.54 (13)
N1—C1—C2	124.36 (16)	N2—C11—C15	109.74 (14)
N1—C1—H1A	117.8	N2—C11—C10	102.72 (13)
C2—C1—H1A	117.8	C15—C11—C10	108.85 (15)
C3—C2—C1	116.18 (15)	N2—C11—C12	111.34 (14)
C3—C2—C7	122.83 (16)	C15—C11—C12	113.15 (15)
C1—C2—C7	120.99 (16)	C10—C11—C12	110.51 (14)
C2—C3—C4	122.28 (15)	C14—C12—C13	109.8 (2)
C2—C3—H3A	118.9	C14—C12—C11	112.33 (16)
C4—C3—H3A	118.9	C13—C12—C11	112.79 (17)
C3—C4—C5	116.13 (14)	C14—C12—H12	107.2
C3—C4—C9	114.27 (14)	C13—C12—H12	107.2
C5—C4—C9	129.60 (14)	C11—C12—H12	107.2
N1—C5—C4	122.43 (15)	C12—C13—H13A	109.5
N1—C5—C6	110.34 (13)	C12—C13—H13B	109.5
C4—C5—C6	127.23 (14)	H13A—C13—H13B	109.5
N2—C6—N3	113.84 (14)	C12—C13—H13C	109.5
N2—C6—C5	126.50 (14)	H13A—C13—H13C	109.5
N3—C6—C5	119.66 (14)	H13B—C13—H13C	109.5
C2—C7—C8	113.25 (16)	C12—C14—H14A	109.5
C2—C7—H7A	108.9	C12—C14—H14B	109.5
C8—C7—H7A	108.9	H14A—C14—H14B	109.5
C2—C7—H7B	108.9	C12—C14—H14C	109.5
C8—C7—H7B	108.9	H14A—C14—H14C	109.5
H7A—C7—H7B	107.7	H14B—C14—H14C	109.5
C7—C8—H8A	109.5	C11—C15—H15A	109.5
C7—C8—H8B	109.5	C11—C15—H15B	109.5
H8A—C8—H8B	109.5	H15A—C15—H15B	109.5
C7—C8—H8C	109.5	C11—C15—H15C	109.5
H8A—C8—H8C	109.5	H15A—C15—H15C	109.5
H8B—C8—H8C	109.5	H15B—C15—H15C	109.5

### Hydrogen-bond geometry (Å, °)

**Table:**

*D*—H···*A*	*D*—H	H···*A*	*D*···*A*	*D*—H···*A*
O1—H1···N2	0.82	1.68	2.4984 (18)	178
N3—H3···O2^i^	0.86	2.10	2.9330 (19)	162

Symmetry codes: (i) −*x*+1, *y*+1/2, −*z*+1/2.
